# Comparative immunohistochemical analysis of inflammatory cytokines in distinct subtypes of Sweet syndrome

**DOI:** 10.3389/fimmu.2024.1355681

**Published:** 2024-03-11

**Authors:** Panjit Chieosilapatham, Teerada Daroontum, Songkiet Suwansirikul, Romanee Chaiwarith, Phichayut Phinyo, Suteeraporn Chaowattanapanit, Charoen Choonhakarn, Salin Kiratikanon, Rujira Rujiwetpongstorn, Napatra Tovanabutra, Siri Chiewchanvit, Mati Chuamanochan

**Affiliations:** ^1^ Division of Dermatology, Department of Internal Medicine, Faculty of Medicine, Chiang Mai University, Chiang Mai, Thailand; ^2^ Division of Immunology, Department of Microbiology, Faculty of Medicine, Chiang Mai University, Chiang Mai, Thailand; ^3^ Department of Pathology, Faculty of Medicine, Chiang Mai University, Chiang Mai, Thailand; ^4^ Division of Infectious Diseases and Tropical Medicine, Department of Internal Medicine, Faculty of Medicine, Chiang Mai University, Chiang Mai, Thailand; ^5^ Center for Clinical Epidemiology and Clinical Statistics, Faculty of Medicine, Chiang Mai University, Chiang Mai, Thailand; ^6^ Department of Family Medicine, Faculty of Medicine, Chiang Mai University, Chiang Mai, Thailand; ^7^ Division of Dermatology, Department of Medicine, Faculty of Medicine, Srinagarind Hospital, Khon Kaen University, Khon Kaen, Thailand; ^8^ Pharmacoepidemiology and Statistics Research Center (PESRC), Faculty of Pharmacy, Chiang Mai University, Chiang Mai, Thailand

**Keywords:** adult-onset immunodeficiency, anti-IFN-γ autoantibody, cytokine expression, immunohistochemical staining, sweet syndrome

## Abstract

**Background:**

A dysregulated immune response has been implicated in Sweet syndrome (SS) pathogenesis; however, cytokine profiles across different conditions associated with SS — including adult-onset immunodeficiency (AOID) due to anti-interferon (IFN)-γ autoantibodies — remain unknown.

**Objective:**

To investigate alterations in inflammatory cytokines in skin lesions of distinct subtypes of SS.

**Methods:**

Skin biopsies were collected from 42 AOID- and 52 non-AOID-associated SS patients and 18 healthy controls. The comparative immunohistochemical study was conducted using monoclonal antibodies against interleukin (IL)-1β, IL-6, IL-17, IFN-γ, and tumor necrosis factor-α on paraffin-embedded sections. The quantitative percentage positivity and intensity were calculated using computer-based image analysis.

**Results:**

The results showed stronger and more diffuse dermal immunoreactivity for IFN-γ and IL-17 in the AOID-associated (*p* < 0.001 and *p* < 0.001, respectively) and non-AOID-associated SS (*p* < 0.001 and *p* < 0.001, respectively) groups. However, no significant differences in the levels of these two cytokines were observed between the AOID- and non-AOID-associated SS groups. Increased expression of IFN-γ together with IL-17 was also noted in almost all subtypes among non-AOID-associated SS.

**Conclusions:**

These results demonstrate that IFN-γ and IL-17 are implicated in immunopathology of all SS subtypes, including AOID-associated SS, despite the presence of anti-IFN-γ autoantibodies.

## 1 Introduction

Sweet syndrome (SS) is characterized by a neutrophil-rich inflammatory infiltration into the skin in the absence of an infection (1). It is frequently associated with systemic diseases — especially hematologic malignancy — inflammatory bowel disease, and immune-mediated rheumatologic disorders (1, 2). Until now, the pathogenic mechanism of SS remains unclear; however, the available evidence suggests that the dysregulated expression of cytokines and growth factors have been implicated in the pathogenesis (1, 3). Clinical evidence has demonstrated an increased production of the major proinflammatory cytokines (interleukin (IL)-1β, IL-6 and IL-8), T helper type 1 (Th1) cytokines (tumor necrosis factor (TNF)-α and interferon (IFN)-γ), and Th17 cytokine (IL-17) in the skin (4) and serum (5, 6) isolated from SS patients. These cytokines directly contribute to neutrophil recruitment and activation as part of the inflammatory response during its pathogenesis (3).

Recent studies have revealed that SS has become a more common cutaneous manifestation reported in adult-onset immunodeficiency (AOID) (7, 8). The presence of potent and neutralizing autoantibodies which act against IFN-γ are recognized as a cause of AOID (9), leading to an increased susceptibility to infections (10). Like other immunocompromised patients, cutaneous infections in AOID can be the result of opportunistic pathogens that rarely cause disease in immunocompetent people, especially non-tuberculous mycobacteria (NTM) (7, 11). Nonetheless, little is known about the cytokine expression across different subtypes of SS. The aim of this study is to determine the immunohistochemical (IHC) expression of inflammatory cytokines in skin biopsies of various forms of SS, including those associated with AOID.

## 2 Materials and methods

### 2.1 Subjects and study designs

Our retrospective study was conducted on archived cutaneous biopsies of patients with SS who were diagnosed by dermatologists at Maharaj Nakorn Chiang Mai Hospital and Srinagarind Hospital over an eight-year period (2012–2020). The diagnosis of SS was reevaluated and confirmed via histological evaluation. Clinical history and relevant data including sex, age, and underlying conditions associated with SS were collected. According to the clinical setting, SS is categorized as classical (idiopathic), malignancy-associated, drug-induced, and AOID-associated. Cases of AOID were diagnosed when a patient met all of the following criteria: (i) they were an adult (over 18 years of age) who presented with disseminated opportunistic infections that were supposed to be from defects in cell-mediated immunity; (ii) the exclusion of other immunocompromised statuses — including human immunodeficiency virus, malignancy, or as a result of receiving immunosuppressive drugs — was confirmed; and (iii) there was a demonstration of the antibodies to IFN-γ using dot enzyme-linked immunosorbent assay (ELISA) (Maharaj Nakorn Chiang Mai Hospital) (12) or via inhibitory ELISA (Srinagarind Hospital) (13). The control group included normal skin tissue samples of amputated specimens from the accidents or individuals who underwent excisions of benign skin tumors presenting with neither clinical nor pathological findings of neutrophilic dermatoses.

### 2.2 Immunohistochemical study

Formalin-fixed paraffin-embedded (FFPE) tissues were sectioned at a thickness of 5 µm and stained with hematoxylin and eosin (H&E) using standard histological laboratory methods. For IHC staining and analysis, FFPE blocks were cut into 5 μm sections and heated at 60°C for 1 h in a dry oven to soften the paraffin. Briefly, the sections were deparaffinized with xylene and rehydrated using graded ethanol in water. Antigen retrieval was performed by CC1 (prediluted, pH 8.0) antigen retrieval solution (Ventana) performed on the Benchmark ULTRA automated slide Stainer for 32 minutes at 37°C. The sections were incubated with primary antibodies, at manufacturer’s recommended dilution for 32 min at 37°C. IHC staining was performed using a Ventana BenchMark ULTRA autostainer using a standard protocol. The following primary antibodies were used: mouse monoclonal anti-human IL-1β [2H12] (sc130323; Santa Cruz Biotechnology, Inc., USA, 1:50 dilution), rabbit polyclonal anti-human IL-6 (ab6672; Abcam, Cambridge, MA, USA, 1:100 dilution), rabbit polyclonal anti-human IL-17A (ab79056; Abcam, 1:100 dilution), rabbit monoclonal anti-human IFN-γ (ab218426; Abcam, 1:50 dilution), and mouse monoclonal anti-human TNF-α [Clone 28401] (mab610, R&D Systems, Minneapolis, MN, USA, 1:100 dilution). The Ultraview Universal DAB IHC detection kit was used for the visualization reaction (12 min), followed by counterstaining with hematoxylin and blue reagent. The slides were gently washed, dehydrated in graded ethanol and xylene, and mounted on coverslips onto microscope slides using a mounting medium. As positive controls for antibodies, human lymph node, lung, and kidney tissues were used to establish staining for IL-1β, IL-6 and IFN-γ, respectively, while human tonsil tissue was used for IL-17 and TNF-α staining.

### 2.3 Image analysis

Digital images of the IHC-stained slides were acquired using an Aperio Scanscope CS2 whole-slide scanner (Leica Biosystems, Nussloch, Germany) interfaced with Aperio ImageScope version 12 software (Leica Biosystems, Wetzlar, Germany). Regarding the histopathological pattern of SS as a dense and diffuse dermal neutrophilic infiltrate, the corresponding dermal region was outlined using the pen tool in Aperio ImageScope software. The optimized positive pixel count algorithm version 9 embedded in the Aperio ImageScope software was used to quantify positive IHC staining as described elsewhere (14). In brief, the algorithm classifying each pixel into negative (n; blue), weak-positive (wp; yellow), medium-positive (p; orange), and strong-positive (sp; red) bins, by a threshold of intensity values: *I*n (total intensity of negative) = (220,255); *I*wp (intensity threshold of weak-positive pixels) = (175,220); *I*p (intensity threshold of medium-positive pixels) = (100,175); *I*sp (intensity threshold of strong-positive pixels) = (0,100), respectively. These values have an inverse association with the stain darkness, where a high intensity represents unstained pixels, while low intensity represents strong-positive pixels (15). Furthermore, the positivity percentage is given by the total number of positive pixels divided by the number of total pixels (negative and positive) in the analyzed area and multiplied by 100 (16). Quantitative image analysis was also performed using the sum of intensity values for all negative, weak-, medium-, and strong-positive pixels (*I*n+*I*wp+*I*p+*I*sp) divided by the number of total pixels (Ntotal), presenting as an intensity score. Two independent blinded observers (P.C. and T.D.) evaluated serial sections. All measurements were repeated at least three times, and the mean values were determined.

### 2.4 Statistical analysis

SPSS version 23.0 (IBM Corp. 2015. Armonk, NY, U.S.A.) and GraphPad Prism (version 8.0; GraphPad Software, San Diego, CA, U.S.A.) were used to analyze the results. Categorical data were analyzed using the chi-square test and are presented as *n* (%). Data were analyzed via a one-way analysis of variance (ANOVA) and Student’s *t* test, as appropriate, and were displayed as mean ± standard deviation (SD). To account for multiple comparisons, the Tukey test was used (17). Values of *p* < 0.05 were considered statistically significant.

## 3 Results

### 3.1 Patient characteristics

A total of 94 biopsy specimens from 94 patients were included in this study. Of these, 42 had AOID-associated SS (23 men and 19 women; age range 28-76), and 52 had SS without underlying AOID (20 men and 32 women; age range 26-83) (**Table 1**). The median age of SS onset was 54.6 ± 8.8 and 56.0 ± 12.3 years among AOID and non-AOID, respectively. Most cases (43/52, 82.7%) of SS without AOID were classical or idiopathic, 13.5% (7/52) were associated with an underlying malignancy, and only two (3.8%) had drug-induced SS. Unsurprisingly, we noted that AOID-associated SS had significantly higher number of cases with a history of NTM infection when compared to non-AOID (92.9% vs 17.3%, *p* < 0.001).

**Table 1:** Baseline characteristics and clinical features of Sweet syndrome with and without adult-onset immunodeficiency.

Characteristics	SS with Non-AOID (*n* = 52) n (%)	SS with AOID (*n* = 42) n (%)	*p*-value*
Female sex	32 (61.5%)	19 (45.2%)	0.115
Age (mean ± SD, range)	56.0 ± 12.3 (26-83)	54.6 ± 8.8 (28-76)	0.538
Clinical forms of SS			
Classical or idiopathic	43 (82.7%)		
Malignancy- associated	7 (13.5%)		
Drug-induced	2 (3.8%)		
NTM infection	9 (17.3%)	39 (92.9%)	**< 0.001**

AOID, adult-onset immunodeficiency; NTM, non-tuberculous mycobacterial infection; SD, standard deviation; SS, Sweet syndrome.

*Analysis of group characteristic differences by Chi-Square or independent sample t-test (age).

Bold indicates significant differences.

### 3.2 Increased expression of IFN-γ and IL-17 in cases of Sweet syndrome

**Figure 1:**
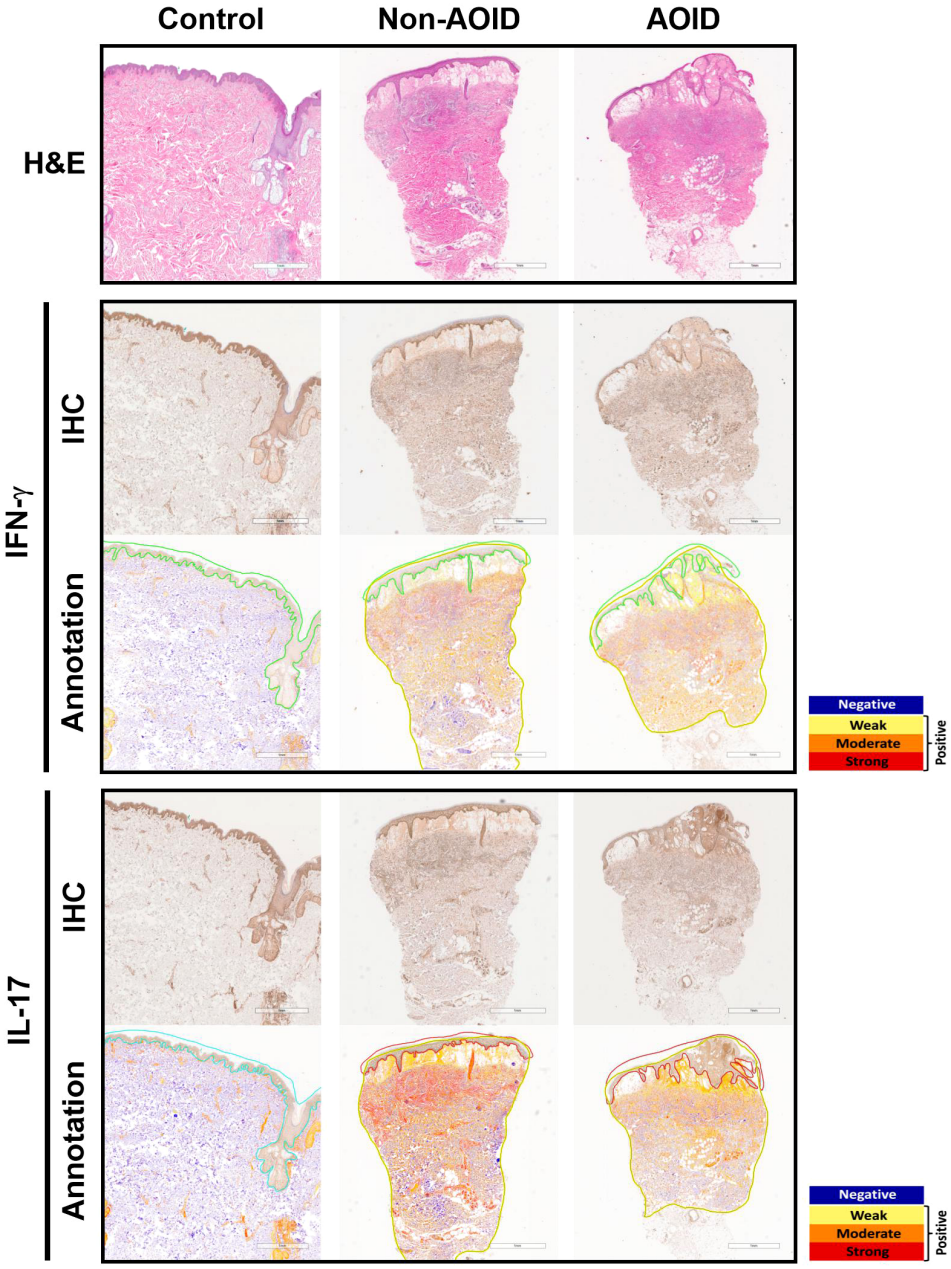
Cytokine expressions in adult-onset immunodeficiency (AOID)-associated Sweet syndrome (SS) is maintained independently despite the presence of anti-interferon (IFN)-γ autoantibodies. Representative images of hematoxylin and eosin (H&E) staining (top panel) in lesion of AOID- and non-AOID-associated SS and control normal tissues Bar = 1 mm. Immunohistochemical (IHC) staining and the corresponding annotated whole slide images for IFN-γ (middle panel) and interleukin (IL)-17 (bottom panel) in lesion of AOID- and non-AOID-associated SS and controls. Bar = 1 mm. A color markup overlay produced by the color deconvolution algorithm reflecting the intensity ranges of image pixels. Blue, negative; yellow, weak positive; orange, medium positive; red, strong positive.

**Figure 2:**
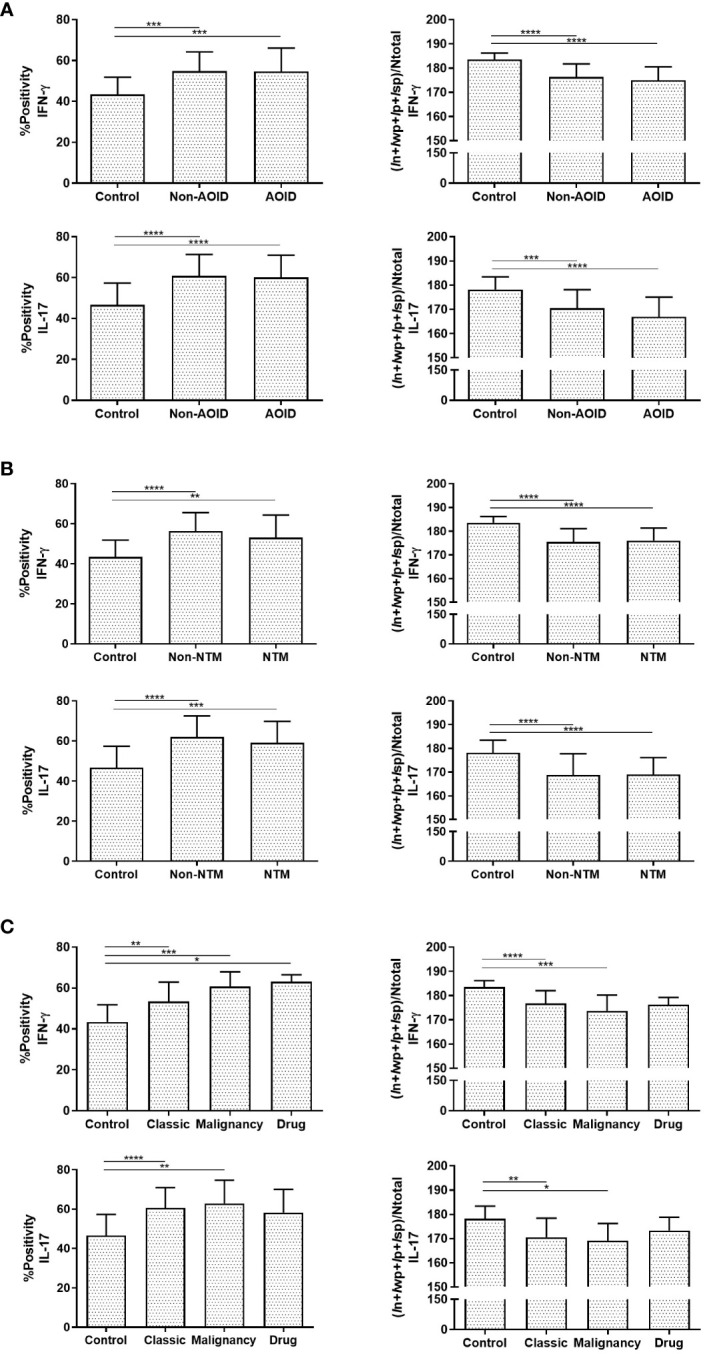
Interferon (IFN)-γ and interleukin (IL)-17 are involved in Sweet syndrome (SS) immunopathology. Graphical representation of percentage of positive (%positivity) and quantification of staining intensity using (*I*n+*I*wp+*I*p+*I*sp)/Ntotal data of IFN-γ and IL-17 among adult-onset immunodeficiency (AOID)- and non-AOID-associated SS and the control normal tissues **(A)**, with or without non-tuberculous mycobacteria (NTM) and control group **(B)**, and with various forms of non-AOID-associated SS **(C)**. Data represent the mean ± SD. *P* values were determined by one-way ANOVA with Tukey adjustments for multiple comparisons where appropriate. Results are expressed in mean ± SD. *P < 0.05, **P < 0.01, ***P < 0.001, ****P < 0.0001. *I*n, total intensity of negative; *I*p, intensity threshold of medium-positive pixels; *I*sp, intensity threshold of strong-positive pixels; *I*wp, intensity threshold of weak-positive pixels; Ntotal, number of total pixels.

All tissue samples from patients with SS — with or without AOID — and controls were examined. Representative images of H&E staining are shown in **Figure 1** (top panels). Marked papillary dermal edema and dense inflammatory cell infiltration, consisting mainly of neutrophils, were evident in SS with and without AOID. Next, we determined the expression of various inflammatory cytokines in the biopsy samples using IHC. Positive IHC staining and the corresponding annotated whole-slide images for IFN-γ (middle panels, **Figure 1**) and IL-17 (bottom panels, **Figure 1**) were observed in diffuse and strong patterns in the dermis of both AOID- and non-AOID-associated SS. Image analysis of IHC staining using the percentage positivity score revealed significantly increased IFN-γ and IL-17 expressions in both AOID-associated SS (*p* < 0.001 and *p* < 0.001, respectively) and non-AOID-associated SS (*p* < 0.001 and *p* < 0.001, respectively) compared to those in the control group (**Table 2**; **Figure 2A**). Similarly, the staining intensity score confirmed significantly increased IFN-γ and IL-17 expressions in both AOID-associated SS (*p* < 0.001 and *p* < 0.001, respectively) and non-AOID-associated SS (*p* < 0.001 and *p* < 0.001, respectively) when compared to those in the controls. The results of quantitative image analysis, as well as the representative images of IHC staining for other cytokines, are presented in **Table 2**; **Supplementary Figure S1**.

**Table 2:** Cytokine expression in Sweet syndrome with and without adult-onset immunodeficiency and controls.

	Controls (*n*=18) Mean ± SD	SS with non-AOID (*n*=52) Mean ± SD	SS with AOID (*n*=42) Mean ± SD	Controls *vs* SS with non-AOID *p-value*	Controls *vs* SS with AOID *p-value*	SS with non-AOID *vs* SS with AOID *p-value*
IFN-γ						
%Positivity	43.4 ± 8.4	54.8 ± 9.5	54.6 ± 11.5	**< 0.001**	**< 0.001**	0.997
(*I*n+*I*wp+*I*p+*I*sp)/Ntotal	183.5 ± 2.6	176.3 ± 5.4	175.0 ± 5.5	**< 0.001**	**< 0.001**	0.432
IL-17						
%Positivity	46.6 ± 10.8	60.8 ± 10.6	60.1 ± 10.8	**< 0.001**	**< 0.001**	0.946
(*I*n+*I*wp+*I*p+*I*sp)/Ntotal	178.2 ± 5.2	170.4 ± 7.7	166.9 ± 8.2	**< 0.001**	**< 0.001**	0.066
IL-1β						
%Positivity	13.1 ± 3.2	12.2 ± 2.9	12.7 ± 4.4	0.628	0.896	0.817
(*I*n+*I*wp+*I*p+*I*sp)/Ntotal	193.4 ± 2.6	191.8 ± 3.1	191.3 ± 4.4	0.220	0.093	0.788
IL-6						
%Positivity	23.8 ± 4.3	26.8 ± 9.3	29.0 ± 12.6	0.557	0.173	0.548
(*I*n+*I*wp+*I*p+*I*sp)/Ntotal	189.8 ± 2.2	186.6 ± 7.4	185.3 ± 7.6	0.231	0.062	0.647
TNFα						
%Positivity	14.3 ± 3.8	12.8 ± 2.6	12.8 ± 3.2	0.184	0.223	0.997
(*I*n+*I*wp+*I*p+*I*sp)/Ntotal	190.7 ± 3.3	191.2 ± 2.4	191.8 ± 2.9	0.788	0.372	0.602

AOID, adult-onset immunodeficiency; IFN-γ, interferon-γ; In, total intensity of negative; IL, interleukin; Ip, intensity threshold of medium-positive pixels; Isp, intensity threshold of strong-positive pixels; Iwp, intensity threshold of weak-positive pixels; Ntotal, number of total pixels; SD, standard deviation; SS, Sweet syndrome; TNF, tumor necrosis factor.

Bold indicates significant differences.

### 3.3 Lack of correlation of cytokine expression associated with non-tuberculous mycobacteria infection

Given that opportunistic infections, and particularly NTM, could be a potential cause of AOID-associated SS by triggering inflammatory conditions (8), we investigated the effects of NTM infection on the expression of various cytokines using IHC analysis. A significant increase in IHC positivity in both IFN-γ and IL-17 in AOID-associated SS with NTM (*p* = 0.003 and *p* < 0.001, respectively) and without NTM (*p* < 0.001 and *p* < 0.001, respectively) was observed compared to the control group (**Table 3**; **Figure 2B**). Similarly, the staining intensity score significantly increased IFN-γ and IL-17 expression in both SS with NTM (*p* < 0.001 and *p* < 0.001, respectively) and without NTM (*p* < 0.001 and *p* < 0.001, respectively) compared to that of the controls. However, no pronounced differences were observed between the NTM-infected and non-NTM-infected groups for any of the cytokines.

**Table 3:** Cytokine expression in Sweet syndrome with and without non-tuberculous mycobacterial infection and controls.

	Controls (*n*=18) Mean ± SD	SS with non-NTM (*n*=46) Mean ± SD	SS with NTM (*n*=48) Mean ± SD	Controls *vs* SS with non-NTM *P-value*	Controls *vs* SS with NTM *P-value*	SS with non-NTM *vs* SS with NTM *P-value*
IFN-γ						
%Positivity	43.4 ± 8.4	56.4 ± 9.2	53.1 ± 11.3	**< 0.001**	**0.003**	0.258
(*I*n+*I*wp+*I*p+*I*sp)/Ntotal	183.5 ± 2.6	175.4 ± 5.6	176.0 ± 5.4	**< 0.001**	**< 0.001**	0.864
IL-17						
%Positivity	46.6 ± 10.8	62.0 ± 10.5	59.1 ± 10.6	**< 0.001**	**< 0.001**	0.404
(*I*n+*I*wp+*I*p+*I*sp)/Ntotal	178.2 ± 5.2	168.8 ± 9.0	168.9 ± 7.1	**< 0.001**	**< 0.001**	0.994
IL-1β						
%Positivity	13.1 ± 3.2	12.3 ± 3.2	12.6 ± 4.1	0.673	0.839	0.920
(*I*n+*I*wp+*I*p+*I*sp)/Ntotal	193.4 ± 2.6	191.7 ± 3.5	191.4 ± 3.9	0.204	0.113	0.921
IL-6						
%Positivity	23.8 ± 4.3	28.6 ± 11.0	27.1 ± 11.0	0.231	0.475	0.786
(*I*n+*I*wp+*I*p+*I*sp)/Ntotal	189.8 ± 2.2	185.3 ± 8.9	186.0 ± 6.0	0.058	0.126	0.865
TNFα						
%Positivity	14.3 ± 3.8	12.7 ± 2.8	12.8 ± 2.9	0.172	0.232	0.970
(*I*n+*I*wp+*I*p+*I*sp)/Ntotal	190.7 ± 3.3	191.5 ± 2.3	191.4 ± 2.9	0.564	0.632	0.987

AOID, adult-onset immunodeficiency; IFN-γ, interferon-γ; In, total intensity of negative; IL, interleukin; Ip, intensity threshold of medium-positive pixels; Isp, intensity threshold of strong-positive pixels; Iwp, intensity threshold of weak-positive pixels; NTM, non-tuberculous mycobacterial infection; Ntotal, number of total pixels; SD, standard deviation; SS, Sweet syndrome; TNF, tumor necrosis factor.

Bold indicates significant differences.

### 3.4 IFN-γ and IL-17 are involved in Sweet syndrome immunopathology

A subtype analysis based on the etiology of SS was also performed to investigate differential cytokine expression through quantitative analysis. In both classic- and malignant-subtypes of non-AOID-associated SS, both IFN-γ (%positivity: *p* = 0.001 and *p* < 0.001, respectively; intensity score: *p* < 0.001 and *p* < 0.001, respectively) and IL-17 (%positivity: *p* < 0.001 and *p* = 0.006, respectively; intensity score: *p* = 0.002 and *p* = 0.031, respectively) were significantly differentially expressed compared with those in controls (**Table 4**; **Figure 2C**). There was a marked increase of IFN-γ in the drug-subtype of non-AOID-associated SS (%positivity: *p* = 0.023).

**Table 4:** Cytokine expression in various forms of non-adult-onset immunodeficiency-associated Sweet syndrome and controls.

	Controls (*n*=18) Mean ± SD	Classic SS (*n*=43) Mean ± SD	Malignancy-associated SS (*n*=7) Mean ± SD	Drug-induced SS (*n*=2) Mean ± SD	Controls *vs* classic SS *P-value*	Control *vs* malignancy-associated SS *P-value*	Control *vs* drug-induced SS *P-value*
IFN-γ							
%Positivity	43.4 ± 8.4	53.4 ± 9.5	60.7 ± 7.3	63.1 ± 4.8	**0.001**	**< 0.001**	0.023
(*I*n+*I*wp+*I*p+*I*sp)/Ntotal	183.5 ± 2.6	176.7 ± 5.3	173.7 ± 6.5	176.2 ± 4.3	**< 0.001**	**< 0.001**	0.197
IL-17							
%Positivity	46.6 ± 10.8	60.6 ± 10.4	62.9 ± 11.8	58.3 ± 16.6	**< 0.001**	**0.006**	0.470
(*I*n+*I*wp+*I*p+*I*sp)/Ntotal	178.2 ± 5.2	170.5 ± 7.9	169.1 ± 7.1	173.4 ± 7.7	**0.002**	**0.031**	0.804
IL-1β							
%Positivity	13.1 ± 3.2	12.5 ± 2.7	11.1 ± 2.7	9.3 ± 8.1	0.895	0.435	0.304
(*I*n+*I*wp+*I*p+*I*sp)/Ntotal	193.4 ± 2.6	191.9 ± 2.9	190.0 ± 4.2	194.2 ± 0.4	0.291	0.051	0.980
IL-6							
%Positivity	23.8 ± 4.3	26.5 ± 9.3	27.5 ± 11.2	28.6 ± 7.6	0.683	0.758	0.870
(*I*n+*I*wp+*I*p+*I*sp)/Ntotal	189.8 ± 2.2	187.0 ± 7.4	183.7 ± 8.0	189.5 ± 5.6	0.445	0.160	0.999
TNFα							
%Positivity	14.3 ± 3.8	13.1 ± 2.6	10.9 ± 1.4	14.0 ± 2.5	0.454	0.053	0.999
(*I*n+*I*wp+*I*p+*I*sp)/Ntotal	190.7 ± 3.3	191.0 ± 2.5	191.7 ± 2.3	193.9 ± 0.5	0.981	0.827	0.389

IFN, interferon; In, total intensity of negative; IL, interleukin; Ip, intensity threshold of medium-positive pixels; Isp, intensity threshold of strong-positive pixels; Iwp, intensity threshold of weak-positive pixels; Ntotal, number of total pixels; SD, standard deviation; TNF, tumor necrosis factor.

Bold indicates significant differences.

## 4 Discussion

The pathogenesis of SS is related to both dysregulated innate and adaptive immune responses, which contribute to the aberration of neutrophil functions (1). Various cytokines that have been implicated in SS pathogenesis are shown in **Supplementary Table S1**, albeit with limited sample sizes and inconsistent results. The analysis from this study, using a larger sample size than previously reported, demonstrates increased expression of IFN-γ and IL-17 in all SS subtypes. Interestingly, these cytokine expression profiles were maintained independently, regardless of the presence of anti-IFN-γ autoantibodies. Similarly, changes in both IFN-γ and IL-17 levels in SS patients with NTM were similar to those in SS patients without NTM.

In line with our study, recent findings strongly support the view that the Th17 axis plays a predominant role in the pathogenesis of SS (1, 3, 18). IL-17 is mainly produced by Th17 cells and is crucial for neutrophil activation and migration through the induction of certain chemokines, including IL-8 and granulocyte colony-stimulating factor (18). In turn, neutrophils can amplify and sustain inflammatory responses by secreting IL-17 (19) as well as Th17 chemoattractants CCL2 and CCL20 (20). Neutrophil-derived IL-17 has been found to regulate IFN-γ production in both autocrine (19) and paracrine (21) manners, contributing to local inflammatory milieu. Interestingly, increased levels of IL-17 in IFN-γ-deficient mice were associated with enhanced neutrophil infiltration at sites of inflammation (22). Furthermore, the presence of anti-IFN-γ autoantibodies had little effect on the production of Th17 cytokines by activated T cells (23).

Th1 responses may be particularly important in the pathogenesis of SS, as shown by the elevated expression of IFN-γ, a signature cytokine of Th1 cells, such as in both lesional skin and serum of SS patients (3, 5). IFN-γ is a multifunctional cytokine secreted by various immune cells, including lymphocytes, natural killer cells, and macrophages (24). Besides its role in Th1 differentiation, IFN-γ could regulate neutrophil functions by the modulating of chemotaxis, phagocytosis, and oxidative burst (24). Despite the presence of neutralizing anti-IFN-γ autoantibodies, we constantly observed significantly elevated expressions of IFN-γ in lesional SS compared with controls. These findings are consistent with previous observations that IFN-γ was upregulated in patients with AOID upon T cell activation (23). Indeed, T-cell response and proliferation could be detected after phorbol myristate acetate and ionomycin stimulation, even in the presence of anti-IFN-γ autoantibodies (23). Increased numbers of neutrophils and natural killer cells in blood samples of AOID may indicate the imbalance of immune system homeostasis (23, 25).

**Figure 3:**
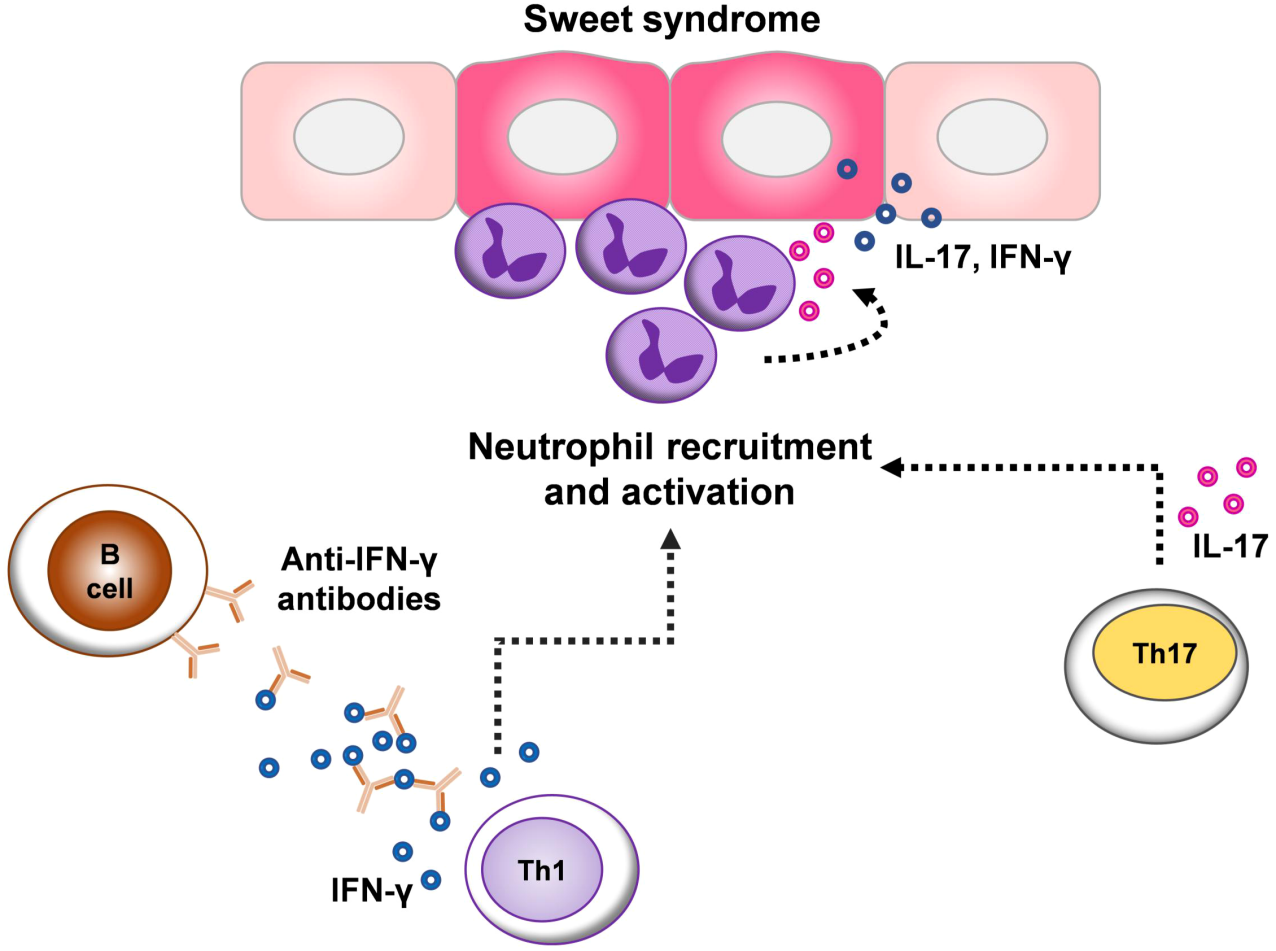
Schematic representation of the proposed cytokine network implicated in the pathogenesis of Sweet syndrome (SS). The pathogenesis of SS is related to both dysregulated innate and adaptive immune responses, which contribute to the aberration of neutrophil functions. T helper (Th) 1 and Th17 responses may be particularly important in the pathogenesis of SS, as shown by the elevated expression of interferon (IFN)-γ and interleukin (IL)-17 in different subtypes of SS. Because IFN-γ and IL-17 could enhance neutrophil infiltration at sites of inflammation and in turn, neutrophils can amplify inflammatory responses though secreting these cytokines contributing to local inflammation. The expression of IFN-γ, despite the presence of anti-IFN-γ autoantibodies in SS lesions indicates an imbalance of immune system homeostasis.

Indeed, diverse antigenic stimuli may provoke a proinflammatory milieu in SS lesions. Dysregulated inflammasome activity can drive the excessive secretion of pro-inflammatory cytokines, especially IL-1β and its downstream target IL-6, leading to enhanced recruitment of neutrophils (26, 27). TNF-α, a major pro-inflammatory cytokine, also promotes neutrophil recruitment to the site of inflammation by regulating endothelial cell activation (28, 29). However, we found no changes in the expression of IL-1β, IL-6, and TNF-α in lesional SS. Several studies have also reported the inhibitory effect of anti-IFN-γ autoantibodies on the production of various cytokines, especially TNF-α (9, 30, 31). These results suggest that the immune response in SS may be mediated by distinct mechanisms (1) (**Figure 3**).

To the best of our knowledge, this study benefits from the recruitment of the largest sample size of all SS subtypes and is the first study to examine inflammatory cytokines in AOID-associated SS. Moreover, the quantitative evaluation of IHC at the tissue level using advanced image analysis techniques, which is contrary to the qualitative or semi-quantitative evaluations in previous reports, is another key strength of our study. However, further studies on additional markers, functional analysis and the regulation of inflammatory processes are needed to confirm and clarify these associations.

In conclusion, our findings confirm the significant participation of IFN-γ and IL-17 in SS lesions, regardless of the presence of anti-IFN-γ autoantibodies.

## Data availability statement

The raw data supporting the conclusions of this article will be made available by the authors, without undue reservation.

## Ethics statement

The study was approved by the Research Ethics Committee of the Faculty of Medicine, Chiang Mai University (MED-2563-07187) and the Khon Kaen University Ethics Committee for Human Research (IRB00001189). The studies were conducted in accordance with the local legislation and institutional requirements. The human samples used in this study were acquired from a by- product of routine care or industry. Written informed consent for participation was not required from the participants or the participants’ legal guardians/next of kin in accordance with the national legislation and institutional requirements.

## Author contributions

PC: Conceptualization, Data curation, Formal analysis, Funding acquisition, Investigation, Methodology, Resources, Software, Validation, Visualization, Writing – original draft. TD: Conceptualization, Data curation, Formal analysis, Investigation, Methodology, Resources, Software, Validation, Visualization, Writing – original draft. SS: Formal analysis, Resources, Software, Visualization, Writing – review & editing. RC: Conceptualization, Data curation, Investigation, Methodology, Resources, Writing – review & editing. PP: Data curation, Formal analysis, Writing – review & editing. SuC: Conceptualization, Data curation, Investigation, Methodology, Resources, Writing – review & editing. CC: Conceptualization, Data curation, Investigation, Methodology, Resources, Writing – review & editing. SK: Conceptualization, Data curation, Investigation, Methodology, Resources, Writing – review & editing. RR: Conceptualization, Data curation, Investigation, Methodology, Resources, Writing – review & editing. NT: Conceptualization, Data curation, Investigation, Methodology, Resources, Writing – review & editing. SiC: Conceptualization, Data curation, Investigation, Methodology, Resources, Writing – review & editing. MC: Conceptualization, Data curation, Formal analysis, Funding acquisition, Investigation, Methodology, Project administration, Resources, Supervision, Validation, Visualization, Writing – review & editing.
