# Supplementary figures and images for: Comparative immunohistochemical analysis of inflammatory cytokines in distinct subtypes of Sweet syndrome
Source: Front Immunol. 2024 Mar 11;15:1355681. doi: 10.3389/fimmu.2024.1355681 (PMC10961367; doi:10.3389/fimmu.2024.1355681)

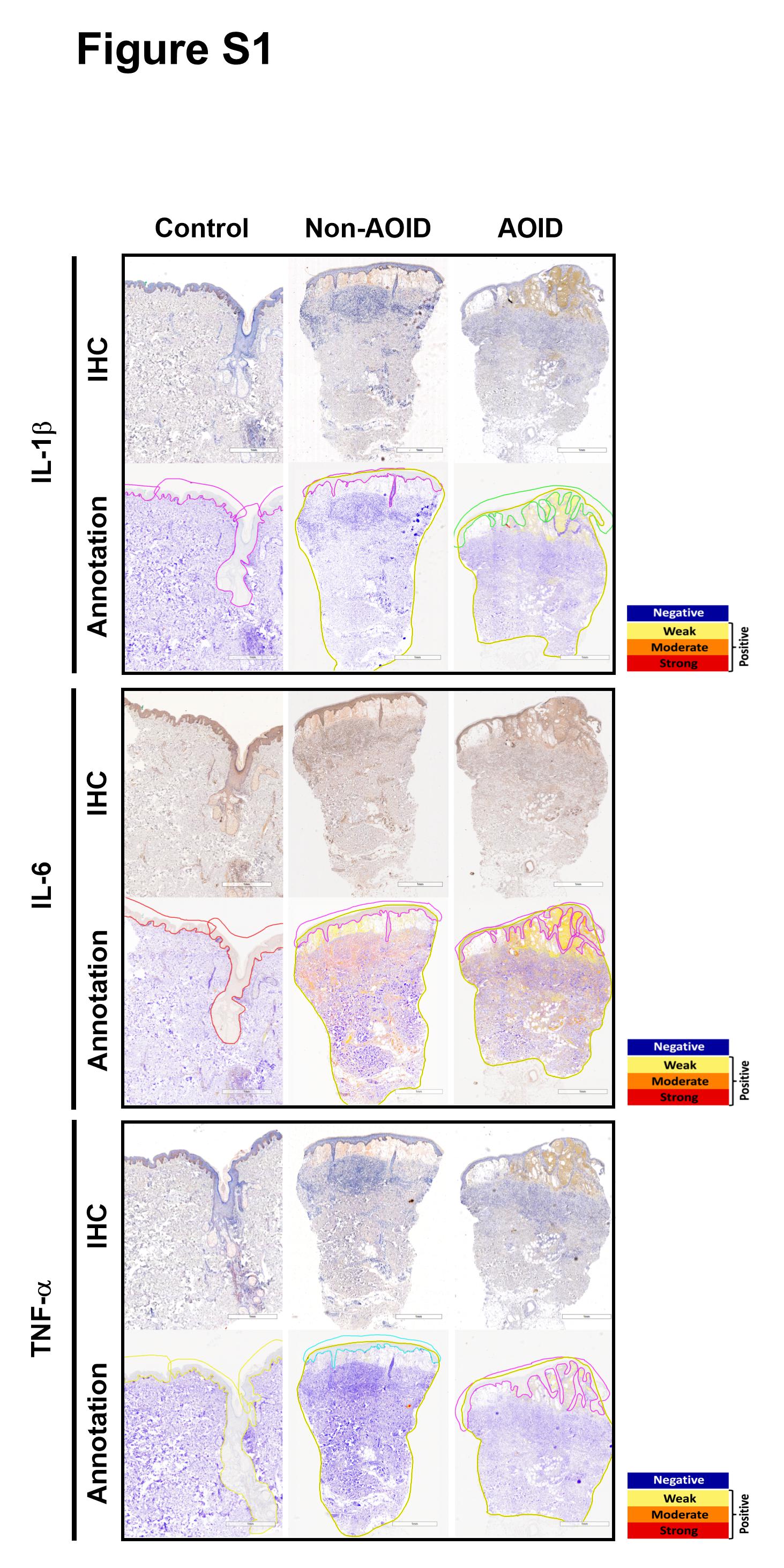

Supplement: Supplementary file 1 [file Image_1.jpg]
