# Supplementary material for: Comparative immunohistochemical analysis of inflammatory cytokines in distinct subtypes of Sweet syndrome
Source: Front Immunol. 2024 Mar 11;15:1355681. doi: 10.3389/fimmu.2024.1355681 (PMC10961367; doi:10.3389/fimmu.2024.1355681)
Supplement: Supplementary file 2 [file Table_1.docx]

Supplementary Material

# Supplementary Tables

## Supplementary Table S1

**Cytokines and signaling molecules in lesional skin and serum in Sweet syndrome**

|  | **Subtypes of SS** | | **IL-1β** | **IL-6** | **IFN-γ** | **TNF-α** | **IL-17** | **Other cytokines** |  |
| --- | --- | --- | --- | --- | --- | --- | --- | --- | --- |
| **Reuss-Borst**  **1993** **(31)** | 1 malignancy-associated (MDS) | | Skin: NA  Serum: ↔ | Skin: NA  Serum: ↑ | Skin: NA  Serum: ↔ | Skin: NA  Serum: ↑ | Skin: NA  Serum: NA | Skin: NA  Serum: ↑G-CSF, GM-CSF |  |
| **Loraas 1994 (32)** | 1 malignancy-associated (MDS) | | Skin: NA  Serum: NA | Skin: NA  Serum: ↑ | Skin: NA  Serum: NA | Skin: NA  Serum: ↔ | Skin: NA  Serum: NA | Skin: NA  Serum: ↔G-CSF, lymphotoxin |  |
| **Giasuddin 1998** **(4)** | 8 classic | | Skin: NA  Serum: ↑ | Skin: NA  Serum: NA | Skin: NA  Serum: ↑ | Skin: NA  Serum: NA | Skin: NA  Serum: NA | Skin: NA  Serum: ↑IL-1α, IL-2  ↔ IL-4 |  |
| **Hattori 2003** **(33)** | 1 malignancy-associated (MDS) | | Skin: NA  Serum: ↔ | Skin: NA  Serum: ↑ | Skin: NA  Serum: NA | Skin: NA  Serum: NA | Skin: NA  Serum: NA | Skin: NA  Serum: ↑G-CSF |  |
| **Kawakami 2004** **(34)** | 12 classic | | Skin: NA  Serum: NA | Skin: NA  Serum: NA | Skin: NA  Serum: NA | Skin: NA  Serum: NA | Skin: NA  Serum: NA | Skin: NA  Serum: ↑G-CSF |  |
| **Uhara 2008 (35)** | 1 malignancy-associated (AML) | | Skin: NA  Serum: NA | Skin: NA  Serum: NA | Skin: NA  Serum: NA | Skin: NA  Serum: NA | Skin: NA  Serum: NA | Skin: NA  Serum: ↑G-CSF |  |
| **Marzano 2010 (3)** | 7 classic  1 malignancy-associated (CLL) | | Skin: ↑  Serum: NA | Skin: NA  Serum: NA | Skin: NA  Serum: NA | Skin: ↑  Serum: NA | Skin: ↑  Serum: NA | Skin: ↑CD3, CD163, IL-8, MMP-2, MMP-9, MPO, VEGF  Serum: NA |  |
| **Marzano 2014 (36)** | 6 classic | | Skin: ↑  Serum: NA | Skin: NA  Serum: NA | Skin: NA  Serum: NA | Skin: ↑  Serum: NA | Skin: ↑  Serum: NA | Skin: ↑CD40L, CXCL-1, -2, -3, -16, Fas, FasL, IL-8, IL-1RI, IL-17R, MMP-2, Siglec-5, -9, TNF-RII, TIMP-1  ↔ MMP-9, RANTES TIMP-2, TNF-RI  Serum: NA |  |
| **Imhof 2015 (37)** | 1 drug-induced (azathioprine) | | Skin: ↑  Serum: NA | Skin: NA  Serum: NA | Skin: NA  Serum: NA | Skin: NA  Serum: NA | Skin: NA  Serum: NA | Skin: NA  Serum: NA |  |
| **Caprony 2015 (38)** | 5 classic | | Skin: NA  Serum: NA | Skin: NA  Serum: NA | Skin: NA  Serum: NA | Skin: NA  Serum: NA | Skin: ↑  Serum: NA | Skin: ↑CD161, IL-10, RORγt, TGF-β  Serum: NA |  |
| **Antiga 2017 (39)** | 5 classic | | Skin: NA  Serum: NA | Skin: NA  Serum: NA | Skin: ↑  Serum: NA | Skin: NA  Serum: NA | Skin: NA  Serum: NA | Skin: ↑CCR3, CCR5, CD40, CD40L, CXCR3, IL-4, IL-5, IL-12, IL-13  Serum: NA |  |
| **Takano 2017 (40)** | 1 classic | | Skin: NA  Serum: ↔ | Skin: NA  Serum: ↑ | Skin: NA  Serum: ↑ | Skin: NA  Serum: ↑ | Skin: NA  Serum: NA | Skin: NA  Serum: ↔IL-18 |  |
| **Fujii 2017** **(41)** | | | 1 classic | Skin: NA  Serum: NA | Skin: NA  Serum: NA | Skin: NA  Serum: NA | Skin: NA  Serum: NA | Skin: NA  Serum: NA | Skin: NA  Serum: ↑G-CSF, amyloid A protein |
| **Matsuzawa 2019** **(5)** | 1 MDS with pulmonary toxoplasmosis | | Skin: NA  Serum: NA | Skin: NA  Serum: ↑ | Skin: NA  Serum: ↔ | Skin: NA  Serum: NA | Skin: NA  Serum: ↔ | Skin: NA  Serum: ↔IL-2 |  |
| **Kusaka 2020 (42)** | 1 neuro-Sweet disease | | Skin: NA  Serum: ↑ | Skin: NA  Serum: ↑ | Skin: NA  Serum: ↔ | Skin: NA  Serum: ↑ | Skin: NA  Serum: ↔ | Skin: NA  Serum: ↑Eotaxin, FGF, G-CSF, IL-1RA, IL-5, IL-7, IL-8, IL-9, IP-10, MCP-1, MIP-1α, -1β, PDGF, RANTES, VEGF  ↔ GM-CSF, IL-2, IL-4, IL-10, IL-12, IL-13, IL-15 |  |
| **Bhattacharya 2023 (43)** | 1 classic | | Skin: ↑  Serum: NA | Skin: NA  Serum: NA | Skin: NA  Serum: NA | Skin: NA  Serum: NA | Skin: NA  Serum: NA | Skin: NA  Serum: ↑PIK3R1 mutation |  |
| **Our study** | 42 AOID  43 classic  7 malignancy-associated  2 drug-induced | | Skin: ↔  Serum: NA | Skin: ↔  Serum: NA | Skin: ↑  Serum: NA | Skin: ↔  Serum: NA | Skin: ↑  Serum: NA | Skin: NA  Serum: NA |  |

*AML*, acute myeloid leukemia; *AOID*, adult-onset immunodeficiency; *CCR*, chemokine receptor; *CD*, cluster of differentiation; *CLL*, chronic lymphocytic leukemia; *CXCL*, C-X-C motif chemokine ligand; *FGF*, fibroblast growth factor; *G-CSF*, granulocyte colony-stimulating factor; *GM-CSF*, granulocyte macrophage colony-stimulating factor; *IFN-γ*, interferon-γ; *IL*, interleukin; *IP-10*, interferon γ-induced protein 10 kDa; *MCP*, monocyte chemoattractant protein; *MDS*, myelodysplastic syndromes; *MIP*, macrophage inflammatory protein; *MPO*, myeloperoxidase; *NA*, not available; *PDGF*, platelet-derived growth factor; *PIK3R1*, phosphotidylinolsitol 3-kinase regulatory subunit 1; *RANTES*, regulated upon activation, normal T cell expressed and presumably secreted; *RORγt*, retinoic acid receptor-related γt orphan nuclear receptor; *SS*, Sweet syndrome; *TIMP*, tissue inhibitor of metalloproteinase; *TNF*, tumor necrosis factor; *VEGF*, vascular endothelial growth factor.
